# The association between hair cortisol and burnout is moderated by age, psychosocial, and immunological markers

**DOI:** 10.1016/j.bbih.2024.100909

**Published:** 2024-11-23

**Authors:** Patrick D. Gajewski, Peter Bröde, Maren Claus, Klaus Golka, Jan G. Hengstler, Carsten Watzl, Edmund Wascher, Stephan Getzmann

**Affiliations:** a Leibniz Research Centre for Working Environment and Human Factors (*IfADo*), Dortmund, Germany; b German Center for Mental Health (DZPG), Partner Site Bochum/Marburg, Germany

**Keywords:** Hair cortisol concentration, Work stress, WAI, Depression, T cells, CD4/CD8 ratio, Proinflammatory cytokines

## Abstract

**Background:**

Exhaustion and depersonalization are the core symptoms of the occupational burnout. However, burnout is not an all-or-nothing phenomenon, but can occur in a milder to moderate form in otherwise healthy employees. In the last two decades hair cortisol concentrations (HCC) were increasingly related to the cumulative effect of psychosocial stress at work. We analyzed data of the Dortmund Vital Study (Clinicaltrials.gov: NCT05155397) to explore the relationship of HCC and burnout symptoms. Moreover, we asked whether the HCC – burnout association was moderated by work ability, chronic stress, neuroticism, depressive symptoms, and stress-related immunological biomarkers such as T cell concentration, CD4/CD8 cell ratio, and proinflammatory cytokines TNF- α, IL-6, and IL-18.

**Methods:**

Burnout was assessed by the Oldenburg Burnout Inventory (OLBI), and the Maslach Burnout Inventory (MBI-D) in 196 working adults aged between 20 and 65 years (mean age 42.2 years). Several self-reported variables and biomarkers were collected.

**Results:**

The results showed an association between HCC and the burnout measures. A series of moderator analyses revealed that the association between HCC and burnout symptoms was substantial for low work ability, high chronic stress level, high neuroticism level, and mild to moderate depressive symptoms. Immunological markers moderated the HCC – burnout association for high concentrations of T cells, low CD4/CD8 ratio and low IL-6, IL-18 and TNF-α concentrations. These interactions were moderated by age showing the largest impact in middle-aged to older individuals.

**Conclusions:**

The present findings shed light on the complex interaction between burnout symptoms and work ability, chronic stress, personality, and the endocrinological and immunological responses across the working lifespan. These parameters should be considered when assessing the risk for developing burnout and validating the diagnosis of burnout.

**Trial registration:**

ClinicalTrials.gov NCT05155397; https://clinicaltrials.gov/ct2/show/NCT05155397.

## Highlights

• Burnout symptoms are associated with hair cortisol concentration (HCC).
•The HCC – burnout axis is moderated by work ability, stress, depression and neuroticism.
• Immunological markers CD4/CD8, T cells, TNF- α, IL-6 and IL-18 moderate the axis.
• The impact of psychosocial and immunological variables on the axis increases with age.
• Significance regions indicate when the moderators significantly affect the axis.

## 1 Introduction

Western societies are affected by the problem of early retirement that stands in contrast to the increased lifespan and often to the statutory pension age. This leads to growing societal costs for retirement and health care (Ilmarinen, 2006). Early retirement is often due to health problems or reduced functional capacity resulting, for example, from high work demands or psychosocial stress (Pilipiec et al., 2020).

Long-term occupational stress due to stressors at work such as qualitative or emotional overload, unclear roles at work, imbalance in work organization, lack of influence at work, or low possibilities for development, low commitment to the workplace, job insecurity, social tension as well as individual factors such as insufficient coping strategies are often preconditions for emotional exhaustion and fatigue (Maslach et al., 2001; Schaufeli et al., 2009). Long-term unfavorable work conditions accompanied by a sense of duty and conscientiousness lead to a perceived discrepancy between personal standards and circumstances in the reality, and a feeling of helplessness and insufficiency. Burnout reflects an end point on the spectrum of such stress-related conditions and is a serious health risk (Ahola et al., 2006; Bianchi et al., 2013).

The prevalence of burnout varies in the literature for specific professions between 0 and 80.5% (Rotenstein et al., 2018, for review). Female workers are more frequently affected, presumably because they often work in professions with numerous and intensive social contacts, such as education and care. There is an ongoing debate in the literature whether burnout and depression are “two sides of the same coin” or distinct diseases with overlapping symptoms. Thus, burnout to date has not been included as an independent mental disorder in the Diagnostic and Statistical Manual of Mental Disorders (DSM-5), while the International Classification of Diseases 11 (ICD-11 World Health Organization, 2022) describes burnout as a syndrome resulting from “chronic workplace stress that has not been successfully managed”. Nevertheless, there are several indications that burnout and depression reflect distinct entities (Ahola et al., 2006; Bärtl et al., 2022; Maslach et al., 2001; Rothe et al., 2020; Tavella et al., 2023) and even show substantial differences in neurocognitive functions (Gajewski et al., 2017a,2017b).

Burnout is commonly measured using the Maslach Burnout Inventory (MBI-GS; Maslach et al., 1986) . The MBI-GS consists of the scales: *emotional exhaustion*, *depersonalization*, and *reduced personal efficacy*. The MBI-GS items for each dimension are framed in the same direction which has been considered as a psychometric shortcoming due to clustering of responses in the negative or positive way. Therefore, Demerouti and colleagues developed an alternative measure of burnout symptoms, the Oldenburg Burnout Inventory (OLBI; Demerouti et al., 2003; 2010). The OLBI includes positively and negatively framed items and evaluates two scales: first, *exhaustion* as consequence of intensive, long-term physical, affective, and cognitive strain, and second, *disengagement from work,* referring to negative attitudes toward the work. The MBI-GS focuses on employees in human services professions, whereas the OLBI can be used in any occupational context (Demerouti et al., 2003).

To improve and prolong work participation and to assess individual and work-related factors, the concept of work ability has been developed (Ilmarinen, 2006). Work ability measured by the Work Ability Index (WAI; Ilmarinen, 2009; Tuomi et al., 1998) is a multifactorial concept referring to an individual's ability to perform the required work. Good work ability results from a balance between physical, mental, emotional, and social abilities on the one hand, and the job demands and work characteristics on the other. Originally, the WAI focused on health and functional capacity of elderly workers, but it can be used in any age group to analyze the status quo and possible changes across the lifespan. Important factors contributing to poor work ability are age, psychosocial stress, neuroticism, low physical activity, obesity, high mental or physical work demands, as well as lack of influence at work (Gajewski et al., 2023; van den Berg et al., 2008). On the other hand, work ability is enhanced by factors like frequent social contacts, high physical fitness, and good quality of life (Rieker et al., 2023).

With regard to the regulation of responses to acute or chronic stress, neuroendocrine hormones are crucial. Particularly the hypothalamus-pituitary-adrenal (HPA) axis is involved in the increase of cortisol level in stress situations (Stalder et al., 2017). However, cortisol levels measured in serum or in saliva vary considerably as function of the daytime and individual and environmental factors, thus providing only short-term information (Dettenborn et al., 2012; McLennan et al., 2016; Rothe et al., 2020). In contrast, long-term cortisol levels indicating permanent high levels of stress are best measured in hairs due to a cumulative secretion over several months (Raul et al., 2004; Stalder and Kirschbaum, 2012; Stalder et al., 2017; Penz et al., 2018a). Recent studies analyzed the association between hair cortisol concentrations (HCC) and long-term stress or even consequences of occupational chronic stress such as burnout or depressive symptoms, but with inconsistent results (Bärtl et al., 2023; Brianda et al., 2020; Kaltenegger et al., 2024, Penz et al., 2018a, 2019; Stalder et al., 2017; Wang et al., 2019; Wekenborg et al., 2019; Wendsche et al., 2020, see Rothe et al., 2020 for review). This could be due to the use of burnout measures with different dimensions and scales such or including populations differing in age, burnout severity or different study designs. For example, Penz and coworkers (Penz et al., 2018a) found a clear positive association between HCC and burnout severity. In contrast, Bärtl et al. (2023) found no association between HCC and burnout in a sample of patients and controls. However, in the burnout group the association was present but only at baseline testing and not after a follow-up measurement seven months later. The authors suggested that HPA axis alterations might become apparent once a particular threshold of burnout has been exceeded.

In addition, long-term stress and resulting burnout affect the immune and metabolic system with upregulation of inflammatory pathways since the HPA axis and the immune system are bidirectionally interwoven (Penz et al., 2018b). Inflammation plays an important role in developing chronic health problems like cardiovascular and metabolic diseases or even cancer (Kaltenegger et al., 2024). Thus, it is important to evaluate metabolic or immune parameters potentially associated with long-term stress and burnout. The association between burnout symptoms and immunological or cardiovascular diseases can be explained using the allostatic load model (McEwen, 1993). This model proposed that disturbances of the physiological system due to chronic stress may increase the risk for physical or mental diseases (allostatic overload) due to imbalance of one or more physiological systems including the immune system, HPA axis, metabolic and cardiovascular systems (Khedri et al., 2020; Juster et al., 2010). This concept is also applicable to the burnout syndrome. Several physiological parameters have been proposed to play an important role in the allostatic overload (Seeman et al., 1997). For example, anthropometric measures such as waist-to-hip ratio (WHR) or body mass index (BMI), cardiovascular parameters such as systolic (SBP) and diastolic blood pressure (DBP), or metabolic parameters such as glycated hemoglobin (HbGlyc), the C-reactive protein (CRP) and HDL-cholesterol levels are predictive for burnout (Bärtl et al., 2022; Kaltenegger et al., 2024). Similar findings were reported for immune parameters such as tumor-necrosis factor-alpha (TNF-α) or Interleukin-6 (IL-6; Penz et al., 2018b). Moreover, other potentially relevant immune parameters that are affected by the hormones released through activation of the HPA axis such as T or NK cells or the CD4/CD8 ratio indicating immunological efficiency were not considered in previous studies on burnout (Khedri et al., 2020 for review).

The present study aims at a possible association between hair cortisol concentration and burnout symptoms in generally healthy working adults from different occupational sectors using two different measures for burnout, OLBI and MBI-D. In a second step, we ask whether the association is moderated by primary outcomes such as work ability, chronic stress at work, neuroticism, and depressive symptoms that play important roles for burnout development, and whether there is an interaction between these factors and the participants’ age. Additionally, we include several potentially relevant, secondary self-reported outcomes such as childhood trauma, stress reactivity, chronotype, cognitive failures in daily life, emotional dissonance, influence at work, job control, physical activity, and quality of life to assess the impact of these variables on the assumed HCC – burnout association in an exploratory way. Finally, we focus on the question whether the HCC – burnout association is moderated by a-priori selected biomarkers of metabolic and immunological functions that may contribute to allostatic overload. In this way, we aim to identify individual markers for the genesis and development of burnout symptoms. Additionally, we provided cutoff scores for the measures that indicate at which level the risk for burnout is significantly increased and which age group is particularly susceptible. This approach could help to shed more light on the complex relationship between chronic stress and the HPA-axis and its consequences for the metabolic and immune system and the experience of exhaustion across the working lifespan.

## 2 Material and methods

### 2.1 Participants

The present analysis was part of the Dortmund Vital Study (DVS, Clinicaltrials.gov: NCT05155397). A total of 199 working individuals volunteered to donate a hair sample with a sufficient length for cortisol analysis. After excluding three outliers (see 2.3), data from 196 participants were included in the analysis. They were between 20 and 65 years of age (*M* = 42.2, *SD* = 12.9 years) and 162 (84.6%) were female. The participants were acquired from a general population in the region of Dortmund and reported no current or past health problems, where “health” was defined in a broad sense and allowed for a history of some diseases such as cardiovascular, immunological, or hormonal health problems. Typical medication such as antihypertensives, blood thinners, cholesterol reducers and hormones did not prohibit participation. There were no restrictions regarding education or occupation. Participants were employed in part-time or full-time jobs and worked in the following sectors: industry (engineering, mechanic, shop fitter, mechatronic, or logistic), service (nurse, social worker, physician assistance, police officer, fire service, hairdressing, waiter, seller, bank assistance, or laboratory assistance), education (teacher, working student, childcare work, or instructor), and craft (automotive mechanic, plumber, electrician, or builder). The characteristics and eligibility criteria of the entire sample were described in the study protocol (Gajewski et al., 2022).

### 2.2 Measurements

In the following the main measures are briefly described. Further details of these measures were published previously (Gajewski et al., 2022, 2023).

#### 2.2.1 Primary self-reported outcomes

Burnout symptoms were assessed using common self-report measures, using the German version of the Maslach Burnout Inventory (MBI-D; Büssing and Perrar, 1992) and the German version of the Oldenburg Burnout Inventory (OLBI; Demerouti et al., 2001). In the present study we computed total scores of the *exhaustion* (9 items) and *depersonalization* (5 items) scales of the MBI-D (6-point Likert-type scale ranging from 1 (never) to 6 (always), total scores: 14–84), and the *exhaustion* scale (8 items) of the OLBI (4-point Likert-type scale ranging from 1 (strongly agree) to 4 (strongly disagree), total scores: 8–32) as dependent variables (Demerouti et al., 2010). Higher scores indicate more severe symptoms. There is no cutoff score for categorizing burnout as it was conceptualized as a continuous phenomenon.

Work stress was assessed by the short scale of chronic stress (SSCS) of the Trier Inventory of Chronic Stress (TICS; Schulz et al., 2004). Neuroticism (emotional instability) was evaluated by the corresponding subscale from the Big Five questionnaire (NEO-FFI; Costa and McCrae, 1992). Depressive symptoms were measured using Beck Depression Inventory (BDI). Work ability was measured with the German version of the Work Ability Index (WAI; Hasselhorn and Freude, 2007). WAI scores range between 7 and 49 points and it is classified into the categories: poor (7–27), moderate (28–36), good (37–43), and excellent (44–49) WA. In the present study the total WAI score was used.

#### 2.2.2 Secondary self-reported outcomes

Further standardized questionnaires were used to measure potential secondary variables: personality traits (Big Five Personality traits, NEO-FFI), traumatic experiences in the childhood (Childhood Trauma Questionnaire, CTQ), chronotype (D-MEQ), cognitive failures in daily life (Cognitive Failure Questionnaire, CFQ), grit personality trait (Grit Scale), emotional dissonance, influence at work and job control, physical activity (Lüdenscheid Physical Activity Questionnaire), and quality of life (WHOQoL-BREF). Stress reactivity was evaluated by the Perceived Stress Reactivity Scale (PSRS), psychosocial stress (Psychosocial Stress Questionnaire, PSQ-20), self-control and self-control at work, chronic stress (Trier Inventory of Chronic Stress, TICS) that consists of several domains: Work Overload, Social Overload, Pressure to Perform, Work Discontent, Demands from Work, Lack of Social Recognition, Social Tensions, Social Isolation, Chronic Worrying).

#### *2.2.3* *Physical fitness test*, *cardiovascular and anthropometric parameters*

Participants’ current physical performance was assessed with the physical work capacity (PWC-130) cycle test, using a bicycle ergometer (Campbell et al., 2001). The aim of this test is to predict the absolute power output at a projected heart rate of 130 beats per minute. Relative power output is calculated by the power-to-weight ratio. In addition, heart rate, electrocardiography (ECG), systolic and diastolic blood pressure were recorded before and during ergometry. Anthropometric parameters like height, weight, waist-to-hip ratio, and Body Mass Index (BMI) were obtained from each participant.

#### 2.2.4 Immunological and metabolic measures

For the analysis of biological parameters, 80 ml of peripheral venous blood was collected. The blood was centrifuged at 4 °C, serum was pipetted and frozen at −80 °C. IL-6, IL-18 and TNF-α were measured using the LEGENDplex™ Human Inflammation Panel 1 from BioLegend, San Diego, CA, United States according to manufacturer's instructions. Cytokines were measured in a subset of n = 102 participants because data of n = 94 participants were not available due to technical problems during the analysis of cytokines. The subgroup with and without cytokine data did not differ regarding age (with: 42.2, without: 42.1 years, *t* < 1), but differed regarding gender distribution (with: 78.4%, without: 90.4% females, *χ*^*2*^ (1) = 5.28, *p* = .022). Further immunological parameters were determined in the whole sample by the analysis of absolute numbers of granulocytes, monocytes, B cells, T cells, Natural Killer (NK) cells and NK-T cells in peripheral blood, and percentages assessing the composition of lymphocyte subsets (Bröde et al., 2023; Claus et al., 2016, for details of the measurements, and Table A1 in the Appendix of Gajewski et al., 2017a for the full list of measured parameters). The CD4/CD8 T cell ratio was transformed to log10-ratio (Bröde et al., 2022). A metric of immune age incorporating high-dimensional ‘omics’ technologies (Alpert et al., 2019) was approximated by an index termed IMMAX (Bröde et al., 2022) calculated from lymphocyte subsets using Principal Component Regression. IMMAX describes a person's immune status independent of chronological age (Bröde et al., 2022, 2023). The concentrations of ammonia, triglycerides, high- and low-density lipoprotein (HDL and LDL), hemoglobin (Hb), C-reactive protein (CRP), and creatinine were measured from peripheral venous blood.

#### 2.2.5 Hair cortisol concentrations

Hair strains were cut close to the scalp from the occipital vertex position. The mean hair length was M = 19.8 (SD = 11.9; min. 3 cm; max. 62 cm). Only participants with a minimum hair length of 3 cm were included. The average hair growth rate is generally assumed with 1 cm/month (Wennig, 2000), the length of 3 cm corresponds to the cumulative glucocorticoid secretion over the 3-month period (Stalder and Kirschbaum, 2012). The hair samples were stored in labeled aluminum foil packages in a dry place and sent for analysis to the LabService GmbH, TU Dresden, Germany. For the cortisol analyses, 7.5 mg of hair was used. HCC was assessed using liquid chromatography-mass spectrometry/mass spectrometry (LC–MS/MS), a standard approach for hair steroid analysis (Gao et al., 2013). HCC results were provided in picograms per milligram (pg/mg). However, the HCC data were positively skewed, and log10-transformation was applied to transform the data to normal distribution (Bärtl et al., 2019, 2023; Wendsche et al., 2020). The log transformed function is unitless.

### 2.3 Data processing and statistical analysis

Three outliers were excluded, defined as above or below 3 standard deviations from the mean of the log10-scaled HCC outcome, leaving n = 196 observations for analysis.

**Fig. 1:**
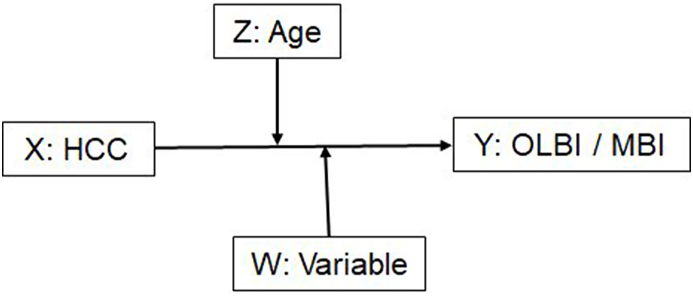
Schematic illustration of the moderation analysis to determine whether the interaction between HCC (X), age (Z), and selected candidate moderator variables (W) significantly predicts severity of burnout symptoms (Y).

In the first step, we computed Pearson correlations between the HCC log10, and the primary and secondary variables grouped in different categories (anthropometric, cardiovascular, metabolic, immunological, personality, stress-related, quality of life). No adjustment of the α level was applied to prevent type 2 errors, i.e., not detecting relevant associations as usually done in exploratory studies (Bender and Lange, 2001; Rothman, 1990). Nevertheless, we additionally provided significant correlations after Bonferroni corrections. In the second step, we conducted moderator analyses as outlined by Fig. 1 using the PROCESS macro for SPSS V. 4.2 (Hayes, 2022), and with HCC used as predictor, OLBI and MBI-D as dependent variables, and Age as well as selected and potentially relevant, primary study variables (WAI, SSCS, Neuroticism, BDI, LDL, T cells, CD4/CD8 ratio, TNF-α, IL-6, IL-18) as moderator variables. The aim was to evaluate whether the relationship between HCC and OLBI or HCC and MBI-D is moderated by age and the primary variables (PROCESS Model 2: Y: OLBI or MBI-D; X: HCC; W: primary variable; Z: Age). We chose Model 2 with two independent moderators because we had no à priori hypotheses about the interaction between age and the primary variables that could moderate the association between HCC and burnout. The analysis elucidates main effects of: HCC, Age, and the selected candidate moderator variable (W) on burnout, the two-way interactions: HCC x Age on burnout, HCC x W on burnout and finally, the three-way interaction HCC x Age x W on burnout. The continuous variable Age was categorized by the moderator analysis in three groups by the mean age (M) and one standard deviation below and above the mean (M ± 1 SD: YA: young adults, 29.2 years; MA: middle-aged adults, 42.0 years; OA: older adults, 54.8 years). After the highest interaction revealed significance the Johnson-Newman intervals provided the range of the moderator variable with significant regression coefficients (Hayes, 2022). These values indicated the significance region for which the HCC – burnout association was substantial for each age group separately. Graphics were provided for the moderator variables with significant interaction X∗W, X∗Z und both interactions. Fig. 1 presents the moderation analysis.

## 3 Results

**Fig. 2:**
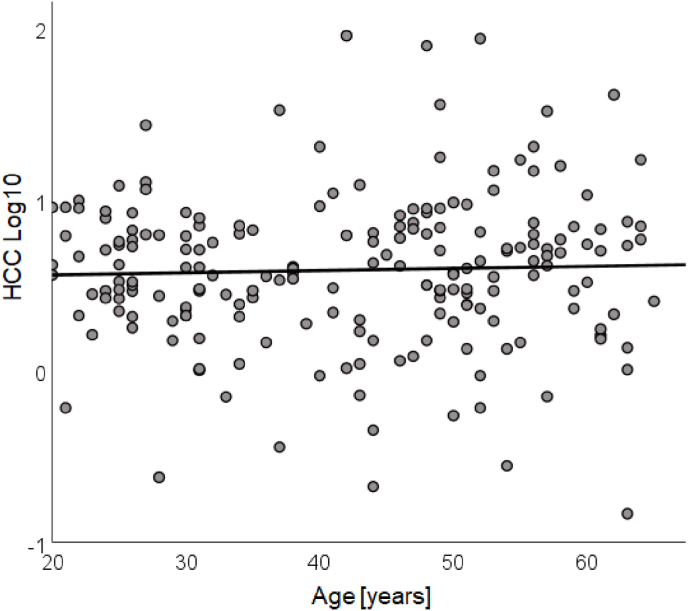
Individual log10-transformed hair cortisol concentration (HCC) as a function of age with the regression line.

The mean concentration of HCC was *M* = 6.84 pg/mg (*SD* = 11.61 pg/mg), with a minimum of 0.15 pg/mg, and a maximum of 90.96 pg/mg. The concentrations are consistent with those reported in other studies that used the LC–MS/MS analytics (Dettenborn et al., 2010; Penz et al., 2019). The mean log10-transformed HCC concentration was *M* = 0.59 (*SD* = 0.44), with a minimum of -0.83, and a maximum of 1.96. The distribution of the log10 HCC as a function of age is presented in Fig. 2.

As burnout and depression are partly overlapping concepts, it was important to analyze the distribution of the depressive symptoms in the sample: n = 183 (84.7%) reported no depression (scores 0–12), n = 23 (10.6%) reported mild depression (13–19), n = 7 (3.2%) reported moderate depression (20–28), and n = 3 (1.4%) reported severe depression. Descriptive statistics of the BDI are included in Table 1.

**Table 1:** Study variables, descriptive statistics, and Pearson correlations with hair cortisol concentration (log10 HCC). Significance level is indicated: **∗***p* < .05, **∗∗***p* < .001. *Italics* indicates substantial correlations after Bonferroni adjustment.

Variable	*M* (*SD*), Range	Correlation with HCC
Age	42.2 (12.9), 20–65	.037
***Anthropometric variables***		
Weight (kg)	74.5 (15.8), 45–145	**.152∗**
Waist circumference (cm)	80.9 (12.5), 56–122	**.151∗**
Hip circumference (cm)	91.0 (12.0), 63–135	**.143∗**
WHR	.88 (.51), .68–1.04	.020
BMI (kg/m^2^)	25.5 (4.8), 18–54	.096
***Cardiovascular variables***		
SBP rest (mmHg)	131.9 (16.1), 90–194	.065
DBP rest (mmHg)	81.8 (9.0), 60–110	.101
HR rest (bpm)	82.3 (12.4), 56–112	.041
SBP max (mmHg)	156.6 (19.7), 110–220	.136
DBP max (mmHg)	93.7 (12.6), 59–151	.044
HR max (bpm)	134.4 (8.9), 93–162	.086
QRS (ms)	90.7 (11.7), 66–148	.006
PWC-130 (W/kg)	1.48 (.44), .40–2.74	.052
PWC-130 max (W)	94.0 (25.4), 36–177	.115
Physical activity (min/week)	361.8 (325.5), 60–2400	.090
***Metabolic variables***		
Hb (g/dL)	13.8 (1.1), 10–17	.122
Creatinine (mg/dL)	.73 (.13), .50–1.30	.066
Cholesterol total (mg/dL)	197.3 (36.5), 108–323	−.082
HDL (mg/dL)	68.9 (19.6), 20–143	***−.281∗∗***
LDL (mg/dL)	123.0 (32.7), 52–227	.012
Triglycerides (mg/dL)	101.9 (48.4), 35–361	**.147∗**
CRP (mg/dL)	1.89 (3.08), .30–28.00	−.008
Ammonia (μg/dL)	23.2 (10.3), 10–57	.107
***Immunological variables***		
Immune age IMMAX	.43 (.10), .17–.82	.125
CD4/CD8 T cells	.53 (.23), .02–1.29	.115
CD4 memory/naive	71.04 (9.97), 20.30–93.00	.069
CD8 memory/naive	22.64 (8.82), 4.81–68.90	−.099
B cells/μl	254.24 (101.88), 24–693	−.105
T cells/μl	1564.36 (509.82), 590–3326	−.030
NK cells/μl	228.17 (116.83), 34–644	.019
NK-T cells/μl	83.13 (76.63), 6–610	−.112
Monocytes/μl	271.01 (118.16), 42–1056	.032
Granulocytes/μl	3988.88 (1359.37), 1750–12518	.001
***Personality variables***		
Neuroticism	19.81 (7.66), 4–47	.072
Extraversion	28.82 (6.73), 7–43	−.050
Openness	30.69 (6.69), 13–46	−.021
Agreeableness	32.43 (5.97), 15–45	.028
Conscientiousness	34.35 (6.68), 8–46	−.011
Self-Control	45.25 (9.30), 10–57	.056
GRIT: Perseverance of effort	3.35 (.55), 1.75–4.63	−.122
DMEQ: Chronotype	54.70 (10.48), 29–75	−.087
***Occupational stress- and job-related variables***		
WAI: Work ability	38.83 (5.57), 16–49	−.015
BDI: Depressive symptoms	6.45 (6.87), 0–40	.134
MBI-D: Emotional exhaustion	31.14 (12.36), 14 - 77	.128
OLBI: Burnout symptoms	16.97 (4.31), 0 -30	***.251∗∗***
CTQ: Childhood trauma	43.46 (13.49), 28 - 90	−.051
Job control	26.20 (6.68), 9 - 36	.009
Self-Control at Work	45.25 (9.30), 23–75	.056
PSQ-20: Psychosocial stress	38.9 (19.2), 0–93.3	**.160∗**
PSRS: Stress reactivity	21.25 (8.43), 0–41	.049
Emotional Dissonance	13.75 (4.54), 5–25	.094
Relaxation	17.26 (4.58), 5–25	−.066
Exhaustion	6.95 (2.73), 0–16	**.143∗**
Recovery	9.91 (3.17), 0–20	**.172∗**
Commitment	17.09 (6.36), 0–28	−.037
Work Overload	13.96 (6.69), 0–32	.133
Social Overload	10.31 (5.22), 0–24	−.004
Pressure to Succeed	15.50 (6.86), 0–33	−.008
Work Discontent	10.13 (5.55), 0–32	.101
Work Demands	5.81 (3.91), 0–20	**.173∗**
Lack of Social Recognition	5.69 (3.77), 0–16	.051
Social Tensions	6.08 (4.24), 0–19	.035
Social Isolation	6.24 (4.67), 0–23	.035
Chronic Worrying	5.81 (3.51), 0 - 16	.126
SSCS: Chronic stress	16.58 (8.69), 0 - 42	**.144∗**
***Quality of Life***		
Physical	16.55 (2.57), 9–20	−.123
Psychological	15.21 (2.23), 7–19	**−.194∗∗**
Social	15.00 (3.14), 4–20	−.105
Environmental	16.35 (1.93), 10–19	−.063
Global quality of Life	15.35 (2.78), 8–20	**−.156∗**

Footnote Table 1.

Abbreviations.

BDI: Beck Depression Inventory.

BMI: Body Mass Index.

CRP: C-Reactive Protein.

CTQ: Childhood Trauma Questionnaire.

DBP: Diastolic Blood Pressure.

D-MEQ: Chronotype questionnaire.

Hb: Hemoglobin (Hb).

HDL: High-Density Lipoprotein.

HR: Heart Rate.

LDL: Low-Density Lipoprotein.

MBI-D: Maslach Burnout Inventory.

OLBI: Oldenburg Burnout Inventory.

PSQ-20: Psychosocial Stress Questionnaire.

PSRS: Perceived Stress Reactivity Scale.

PWC-130: Physical Work Capacity at 130 bpm.

QRS: QRS Complex of the electrocardiogram.

SBP: Systolic Blood Pressure.

WHR: Waist-To-Hip ratio.

Descriptive statistics and Pearson correlations with the log10-transformed HCC for all measures constituting the 7 variable categories are presented in Table 1. As evident from Fig. 2 and Table 1 there was no significant correlation between HCC and the age of the participants.

### 3.1 Regression analyses for HCC and OLBI and HCC and MBI-D

**Fig. 3:**
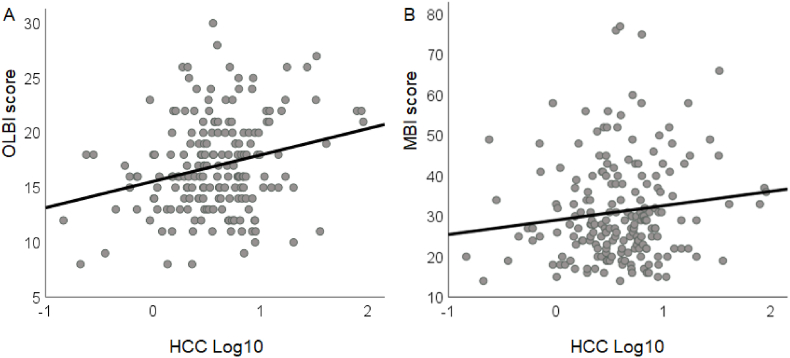
Individual scores in OLBI (A) and MBI-D (B) as function of log10-transformed hair cortisol concentration (HCC) with regression lines.

Univariate regression analyses were applied to test if HCC predicted participants' severity of burnout symptoms measured with OLBI and MBI-D (Fig. 3). The results indicated that the HCC predicted OLBI outcome (*R*^*2*^ = .063, *F*(1,187) = 12.46, *p* < .001), while MBI-D showed a trend (*R*^*2*^ = .016, *F*(1,193) = 3.17, *p* = .076).

### 3.2 Correlations between study variables and HCC

The analyses presented in Table 1 revealed positive correlations between anthropometric variables (weight, waist and hip circumference) and HCC, while WHR and BMI were not correlated with HCC. Regarding metabolic variables, there was a negative correlation with high-density lipoprotein and a positive correlation with triglycerides. None of the measured immunological parameters correlated with HCC.

OLBI was positively correlated with HCC, but MBI-D was not. Occupational and stress-related variables such as psychosocial stress, exhaustion, lack of recovery, high work demands, and chronic stress were positively correlated with HCC. Finally, the self-reported psychological and general quality of life were negatively correlated with log10 HCC. It is important to note that no adjustment of the α level was applied because of an explorative type of the analysis. After Bonferroni adjustment, the corresponding α level is *p <* .05/71 = .0007, suggesting that only the correlations of HCC with HDL and OLBI remained significant after the correction.

### 3.3 Moderation analyses for the primary variables: work ability, chronic stress, depressive symptoms, neuroticism, and age predicting the association between HCC and burnout

To evaluate the interaction of Age and primary self-reported variables on the relationship between HCC and burnout symptoms, moderation analyses were carried out for OLBI and MBI-D as dependent variables.

#### 3.3.1 Work ability (WAI)

**Fig. 4:**
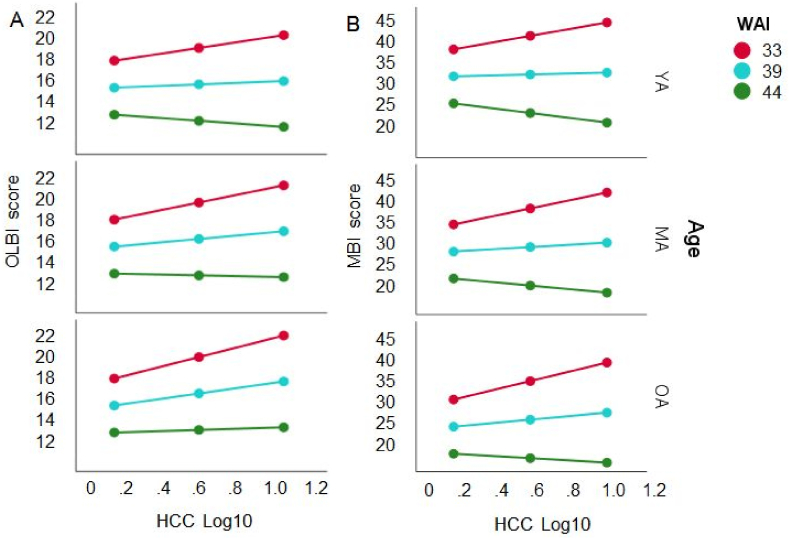
The linear association between log 10 HCC and OLBI (left panel A) and MBI-D (right panel B) is moderated by WAI (*M* ± 1 *SD*: red = low, blue = intermediate, green = high) and age of the working participants (*M* ± 1 *SD*: YA = young adults, MA = middle-aged adults, OA = older adults). The HCC – OLBI association is stronger with decreasing work ability and increasing age. (For interpretation of the references to colour in this figure legend, the reader is referred to the Web version of this article.)

For OLBI, the overall model was significant (*F*(5, 182) = 18.16, *p* < .0001), predicting *R*^*2*^ = 33.29% of the variance. The analysis indicated a small main effect of WAI on OLBI (*t* = −1.964, *p* < .05), indicating that lower work ability was associated with higher OLBI scores and no effect of Age on OLBI was found (*t* = −1.406, *p* = .163). Importantly, WAI moderated the HCC – OLBI association significantly (Δ*R*^*2*^ = 3.21%, *F*(1, 182) = 8.76, *p* < .01), but Age did not (Δ*R*^*2*^ = 0%; *F*(1, 182) < 1). Moreover, WAI and Age simultaneously moderated the HCC – OLBI association (Δ*R*^*2*^ = 3.32%; *F*(2, 182) = 4.53, *p* = .012). This illustrates Fig. 4a, suggesting that the HCC – OLBI association differed for different scores of the WAI and Age: for low work ability (WAI scores lower than 33.2), the association was significant for all age groups (younger: *t* = 3.279, *p* < .01; middle-aged: *t* = 4.861, *p* < .001; older adults: *t* = 4.528, *p* < .001). For medium work ability (WAI scores between 33.2 and 38.8), the interaction was significant for middle-aged (*t* = 2.788, *p* < .01) and older participants (*t* = 2.175, *p* < .05), but not for younger ones (*t* = 1.766, *p* = .078). No moderation of the HCC – OLBI association was found for high work ability (WAI scores larger than 40; *t* < 1).

A similar pattern was found for MBI-D. The overall model was significant (*F*(5, 188) = 18.68, *p* < .0001), predicting a large portion of the variance (*R*^*2*^ = 33.2%). The analysis yielded an effect of WAI (*t* = −2.932, *p* < .01) and Age (*t* = −2.554, *p* < .05) on MBI-D. There was an interaction with WAI (Δ*R*^*2*^ = 2.0%, *F*(1, 188) = 5.59, *p* < .05), but not with Age (Δ*R*^*2*^ = .01%, *F*(1, 188) < 1). Age and WAI moderated the HCC – MBI-D association (Δ*R*^*2*^ = 2.4%, *F*(2, 188) = 3.31, *p* < .05). As evident from Fig. 4b the pattern was similar to that in Fig. 4a with WAI cutoff score lower than 38.0 being significant for middle-aged (*t* = 2.935, *p* < .01) and older participants (*t* = 3.161, *p* < .01), indicating that the association is not significant for good work ability. In other words, the association between HCC and burnout symptoms showed a more positive slope with increasing age and decreasing work ability.

#### 3.3.2 Chronic stress (SSCS)

**Fig. 5:**
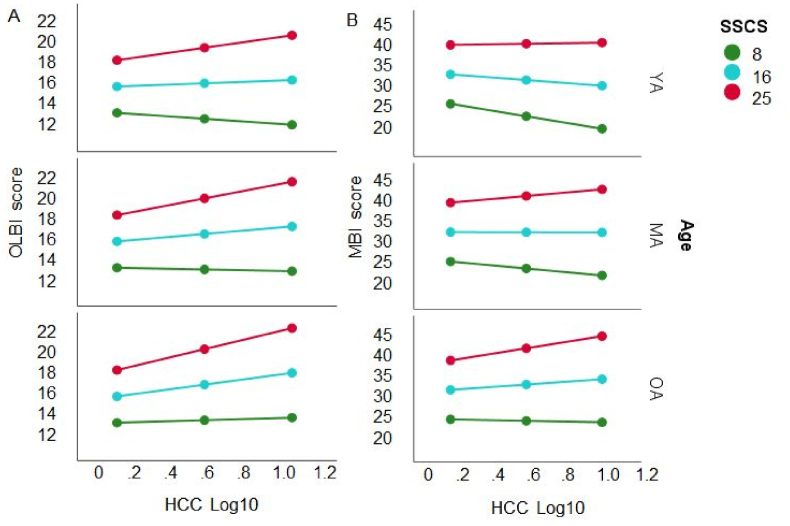
The linear association between HCC and OLBI (left panel A) and MBI-D (right panel B) is moderated by SSCI scores (*M* ± 1 *SD*: red = high, blue = intermediate, green = low) and age of the participants (*M* ± 1 *SD*: YA = young adults, MA = middle-aged adults, OA = older adults). The association between HCC and OLBI showed a more positive slope with increasing chronic stress and age. (For interpretation of the references to colour in this figure legend, the reader is referred to the Web version of this article.)

A moderation analysis was carried out to determine whether the interaction between the independent factors chronic stress (SSCS) and Age moderates the HCC – OLBI association. The overall model was significant (*F*(5, 181) = 28.58, *p* < .0001), explaining *R*^*2*^ = 44.1% of the variance. The analysis yielded an effect of SSCS on OLBI (*t* = 5.196, *p* < .0001). Again, no effect of Age on OLBI was found (*t* < 1). However, there was an interaction of SSCS and HCC (Δ*R*^*2*^ = 3.4%, *F*(1, 181) = 10.92, *p* < .01) and of SSCS and Age (Δ*R*^*2*^ = 3.5%, *F*(2, 181) = 5.64, *p* < .01), indicating a substantial relationship HCC – OLBI in young (*t* = 2.164, *p* < .05), middle-aged (*t* = 4.074, *p* < .001) and older working adults (*t* = 2.935, *p* < .01) with SSCI stress scores larger than 25, and less strong, but significant association with SSCI scores ranging between 16 and 25 in middle-aged (*t* = 2.213, *p* < .05) and older working adults (*t* = 2.800, *p* < .01), but not in the younger ones (*t* < 1). With SSCS scores lower than 16 there was no significant HCC – OLBI association as evident in Fig. 5a. Taken together, the association between HCC and OLBI showed a more positive slope with increasing chronic stress and age.

For the MBI-D the overall regression model was significant (*F*(5, 187) = 30.42, *p* < .0001), explaining *R*^*2*^ = 44.9% of the variance. There was an effect of SSCS on MBI-D (*t* = 6.372, *p* < .0001). No effect of Age on MBI-D was found (*t* < 1). Moreover, there was an interaction of SSCS and HCC (Δ*R*^*2*^ = 2.03%, *F*(1, 187) = 6.89, *p* < .01), and of SSCS, HCC and Age (Δ*R*^*2*^ = 2.4%, *F*(2, 187) = 4.15, *p* < .05) on MBI-D. However, the HCC – MBI-D association was only substantial in older adults with the highest levels of stress (*t* = 2.525, *p* < .05, Fig. 5b bottom panel).

#### 3.3.3 Depressive symptoms (BDI)

Further analysis showed that depressive symptoms assessed using the BDI significantly moderated the HCC – OLBI association. Again, the model was significant (*F*(5, 182) = 20.34, *p* < .0001), predicting *R*^*2*^ = 35.8% of the variance. The analysis indicated an effect of BDI (*t* = 3.87, *p* < .001), and a significant interaction of BDI and HCC on OLBI (Δ*R*^*2*^ = 1.6%, *F*(1, 182) = 4.55, *p* < .05). No effect of Age or interaction with BDI x Age on OLBI was found (both *F*-values < 1). The analysis showed a trend for a simultaneous moderation of the HCC – OLBI association by Age and BDI (*F*(2, 182) = 2.58, *p* = .078). Conditional effects revealed however, that the HCC – OLBI association was significantly moderated for BDI scores higher than 7 (*p* < .05 to *p* < .01), indicating mild to moderate depression. This was evident in the middle-aged and the older group.

For the MBI-D, the overall model was significant (*F*(5, 188) = 18.79, *p* < .0001), explaining *R*^*2*^ = 33.3% of the variance. There was an effect of BDI (*t* = 4.328, *p* < .0001), but the interactions of BDI and HCC (*F*(1, 188) = 3.34, *p* = .068), or of BDI, Age, and HCC (*F*(1, 188) = 2.35, *p* = .098) did not reach significance.

#### 3.3.4 Neuroticism (NEO-FFI)

**Fig. 6:**
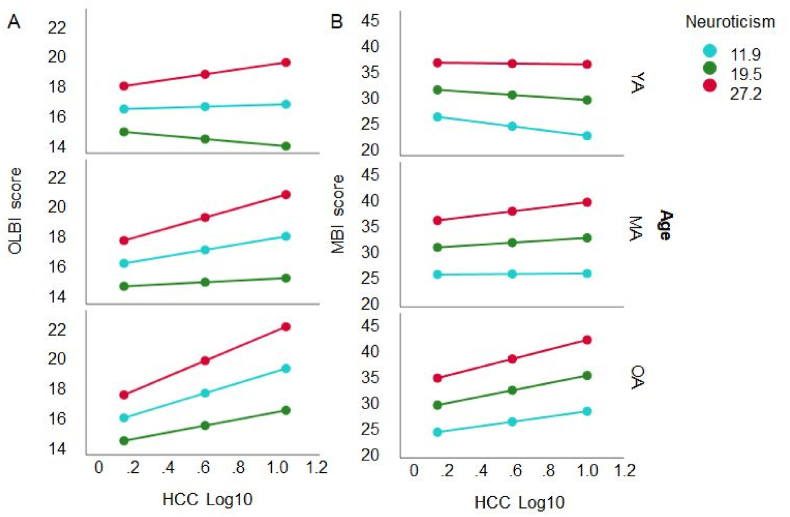
The linear association between HCC and OLBI (left panel A) and MBI-D (right panel B) is moderated by the neuroticism score (*M* ± 1 *SD* of the total score: red = low, blue = intermediate, green = high) and Age of the adults (*M* ± 1 *SD*: YA = young adults, MA = middle-aged adults, OA = older adults). The association HCC-OLBI increases as a function of increased neuroticism score in MA and even more in OA.

A final analysis for primary self-reported outcomes was run to determine whether the interaction between the Neuroticism and Age moderated the HCC – OLBI association. The model was significant (*F*(5, 182) = 14.8, *p* < .0001), predicting *R*^*2*^ = 28.9% of the variance. The analysis indicated an effect of Neuroticism (*t* = 2.53, *p* < .05), and a significant interaction of Neuroticism and HCC on OLBI (Δ*R*^*2*^ = 2.4%, *F*(1, 182) = 6.10, *p* < .05). No effect of Age (*t* < 1), but a trend for interaction HCC and Age on OLBI was observed (*F*(1, 182) = 3.49, *p* = .063). Importantly, the HCC – OLBI association was simultaneously moderated by Age and Neuroticism (*F*(2, 182) = 3.97, *p* < .05). Conditional effects revealed that the HCC – OLBI association was significantly moderated for mean neuroticism scores higher than 19 in middle-aged and older working adults (*p* < .01 to *p* < .0001), indicating a stronger association at higher neuroticism scores and older age (Fig. 6a).

For MBI-D the model was significant (*F*(5, 188) = 13.4, *p* < .0001), predicting *R*^*2*^ = 26.3% of the variance. There was an effect of Neuroticism on MBI-D (*t* = 3.94, *p* < .0001), and a significant interaction of Age and HCC on MBI-D (*F*(1, 188) = 4.23, *p* < .05, Δ*R*^*2*^ = 1.7%). The interaction of Neuroticism, Age and HCC on MBI-D did not reach significance (*F*(2, 188) = 2.35, *p* = .098, Fig. 6b).

### 3.4 Moderation analysis on the interaction of immunological biomarkers and age predicting the association between HCC and burnout

#### 3.4.1 T cells

**Fig. 7:**
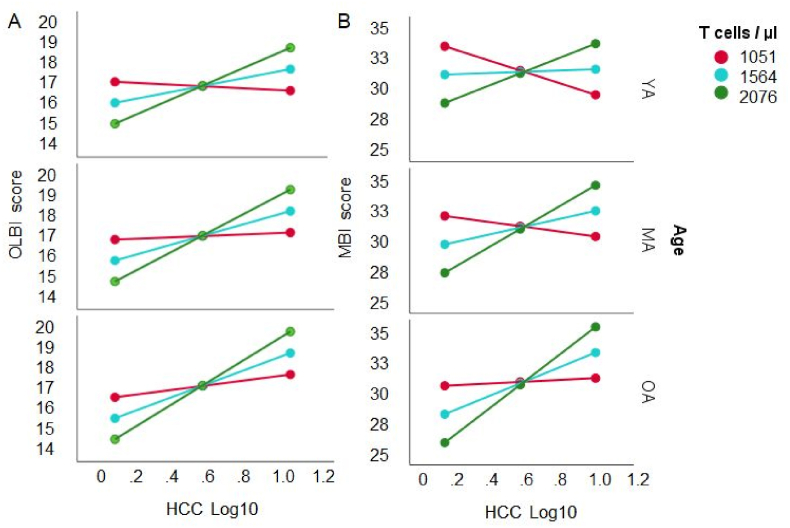
The linear association between HCC and OLBI (left panel A) and MBI-D (right panel B) is moderated by T cell concentrations (*M* ± 1 *SD* number per μl serum: red = low, blue = intermediate, green = high) and Age of the working adults (*M* ± 1 *SD*: YA = young adults, MA = middle-aged adults, OA = older adults). The associations HCC-OLBI and HCC-MBI-D inversed as a function of T cell concentrations. (For interpretation of the references to colour in this figure legend, the reader is referred to the Web version of this article.)

For the T cell concentration and Age the regression model was significant (*F*(5, 176) = 4.13, *p* < .01), explaining *R*^*2*^ = 10.5% of the variance. There was an effect of T cell concentration on OLBI (*t* = −2.214, *p* = .028). No effect of Age was found (*t* < 1). Moreover, there was an interaction of T cell concentration and HCC (Δ*R*^*2*^ = 3.50%, *F*(1, 176) = 6.88, *p* = .0095) and of T cell concentration, HCC, and Age (Δ*R*^*2*^ = 3.74%, *F*(2, 176) = 3.68, *p* = .0271) on OLBI. As evident from Fig. 7a, conditional effects showed that the regression for the HCC – OLBI association was significant for T cell concentrations of more than 1553 cells per μl in participants older than 42 years (*p* < .01 to *p* < .001).

The overall regression model for MBI-D did not reach significance (*F*(5, 182) = 1.80, *p* = .114), but the effect of T cell concentration did (*t* = −1.992, *p* < .05). No effect of Age was found (*t* < 1), but there was an interaction of T cells and HCC (Δ*R*^*2*^ = 2.65%, *F*(1, 182) = 5.06, *p* < .05), and of T cells, HCC, and Age (Δ*R*^*2*^ = 3.14%, *F*(2, 182) = 3.00, *p* < .05). Here, the conditional effect was less consistent and significant for concentrations higher than 2076 per μl in participants older than 42 years (Fig. 7b).

#### 3.4.2 CD4/CD8 T cell ratio

**Fig. 8:**
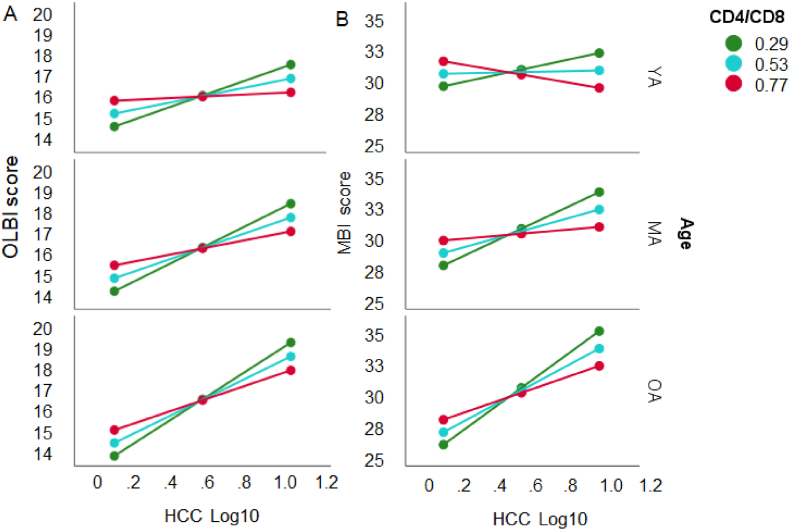
The linear association between HCC and OLBI (left panel A) and MBI-D (right panel) is moderated by log10 transformed CD4/CD8 T cells ratio (*M* ± 1 *SD*: red = high, blue = intermediate, green = low) and Age of the working adults (*M* ± 1 *SD*: YA = young adults, MA = middle-aged adults, OA = older adults). (For interpretation of the references to colour in this figure legend, the reader is referred to the Web version of this article.)

For OLBI, the overall model with the log transformed CD4/CD8 ratio and Age reached significance (*F*(5, 181) = 3.55, p < .01), explaining 9.1% of the variance. No effects or interactions with Age on OLBI were found (*t* < 1). However, there was an interaction of the CD4/CD8 ratio with HCC (*F*(5, 180) = 4.30, *p* < .05), showing a moderation of the relationship HCC – OLBI for low ratios of CD4/CD8 in all participants groups (*p* < .05 to *p* < .001), in middle-aged and older participants for intermediate ratios (*p* < .001), and for highest ratios in older adults only (*p* < .05), although the interaction with Age did not reach significance (*F*(2, 181) = 2.45, *p* = .092, Fig. 8a). MBI-D did not show the corresponding pattern and the overall model was not significant (*F*(5, 186) = 1.40, *p* = .225, Fig. 8b).

#### 3.4.3 TNF-α

For the *TNF-α* concentration in serum and Age the regression model was significant (*F*(5, 90) = 2.40, *p* < .05), explaining *R*^*2*^ = 11.13% of the variance. There was an effect of Age on OLBI (*t* = −2.492, *p* < .05). No effect of TNF-α on OLBI was found (*t* = 1.50, *p* = . 136). Notably, there was an interaction of TNF-α, Age, and HCC (Δ*R*^*2*^ = 7.9%, *F*(2, 90) = 4.03, *p* < .05). The conditional effects showed that the regression for the HCC – OLBI association was significant for TNF-α concentrations below 4.2 pg/ml in older participants (*p* < .01 to *p* < .01).

For the MBI-D, the pattern was similar, however the overall model did not reach significance (*F*(5, 94) = 1.57, *p* = .17).

#### 3.4.4 IL-6

For the IL-6 concentration in serum and Age the regression model showed only a trend (*F*(5, 90) = 2.17, *p* = .064), *R*^*2*^ = 10.8%. There was an effect of Age (*t* = −2.620, *p* < .01) and HCC on OLBI (*t* = −2.096, *p* < .05). No effect of IL-6 was found (*t* < 1). However, there was an interaction of IL-6, Age, and HCC on OLBI (Δ*R*^*2*^ = 6.3%, *F*(2, 90) = 3.165, *p* < .05). The conditional effects showed that the regression for the HCC – OLBI association was significant for lower IL-6 concentrations below 12.9 pg/ml in participants older than 54 years (*p* < 01 to *p* < 05). For MBI-D the overall model was not significant (*F*(5, 94) = 1.45, *p* = .214), *R*^*2*^ = 7.2%.

#### 3.4.5 IL-18

**Fig. 9:**
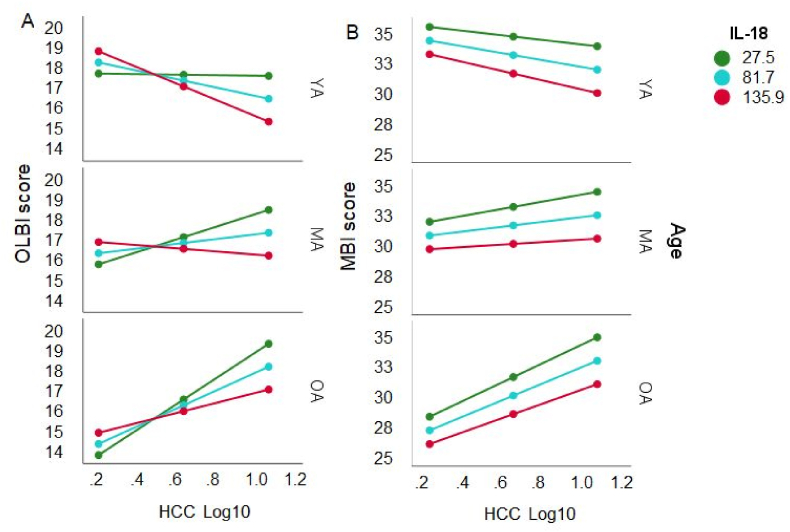
The linear association between HCC and OLBI (left panel A) and MBI-D (right panel) is moderated by IL-18 concentration in pg/ml (M ± 1 SD: red = high, blue = intermediate, green = low) and Age of the working adults (M ± 1 SD: YA = young adults, MA = middle-aged adults, OA = older adults). Please note the change of direction of the association between the age groups and IL-18 concentrations. (For interpretation of the references to colour in this figure legend, the reader is referred to the Web version of this article.)

For the IL-18 concentration the overall regression model predicting the HCC – OLBI association was significant (*F*(5, 90) = 2.98, *p* < .05), *R*^*2*^ = 14.2%. There was an effect of Age (*t* = −2.23, *p* < .01). No main effect of IL-18 was found (*t* < 1). However, there were interactions of IL-18 and HCC (Δ*R*^*2*^ = 4.7%, *F*(1, 90) = 4.965, *p* < .05), and of IL-18, Age, and HCC (Δ*R*^*2*^ = 10.6%, *F*(2, 90) = 5.546, *p* < .01). The conditional effects show that the regression for the HCC – OLBI association was significant for IL-18 concentrations of 81.7 pg/ml in older participants (*p* < .0001 to *p* < .05), for concentrations of 27.5 pg/ml in middle-aged group (*p* < .05), and for concentrations of 135.9 pg/ml in young adults (*p* < .05) as illustrated in Fig. 9a but the direction of the association changed between the age groups, showing a negative relationship in young age for high concentrations and a positive association in older adults.

For MBI-D the regression model was not significant (*F*(5, 94) = 2.02, *p* = .082), *R*^*2*^ = 9.7%).

Finally, the HCC – OLBI association was not substantially moderated by metabolites and Age: Hb, CRP, triglycerides, or ammonia (all *F*-values < 1), and HDL (*F*(2,185) = 1.95, *p* = .16), or further immunological parameters: monocytes, B cells, NK cells (all *F*-values < 1), granulocytes (*F*(2,176) = 1.62, *p* = .19), and NK-T cells (*F*(2,176) = 1.97, *p* = .14).

## 4 Discussion

The present study aimed to examine the association between hair cortisol concentrations (HCC) and burnout symptoms assessed by two different measures, the OLBI and the MBI-D. Moreover, correlations of HCC and several anthropometric, cardiovascular, metabolic, immunological, psychosocial, personality and lifestyle variables were tested. The second aim of the study was to analyze which of the primary variables such as work ability, chronic stress, neuroticism or depressive symptoms known to be important for genesis and development of burnout moderate the association between HCC and burnout. These analyses allow for detecting the cutoff levels of stress, neuroticism, depression, or work ability exhibiting a substantial influence on the association between HCC and burnout. Furthermore, we asked which biological markers contributing to the empirical model of allostatic (over)load (Juster et al., 2010; McEwen, 1993) moderate the relationship between HCC and burnout. For this reason, a series of moderation analyses were conducted with age and immunological variables to identify the most influencing biomarkers.

First, and most importantly we observed a substantial association between HCC and OLBI as a measure of exhaustion, the main component of burnout, thus corroborating previous findings (Penz et al., 2018a; Stalder et al., 2017; Wang et al., 2019; Wendsche et al., 2020), in contrast to other recent findings (Bärtl et al., 2023; Kaltenegger et al., 2024; Rothe et al., 2020 for review). The association between HCC and MBI-D was less evident.

### 4.1 Correlational analyses

Correlational analyses showed positive associations between hair cortisol and weight, waist, and hip circumferences (but not WHR or BMI), confirming earlier reports that showed that long-term overactivation of the HPA axis is related to overweight and obesity (Kaltenegger et al., 2024; Rothe et al., 2020; Stalder et al., 2017) but it was surprising that BMI or WHR were not correlated with the HCC. However, BMI is computed on the parameters body weight and height, but BMI does not differ between muscle mass and fat mass. Therefore, these results provide further evidence that BMI is a crude measure that should be supplemented by further parameters, e.g., waist and hip circumferences. Measures of body fat have a stronger correlation with inflammatory markers than BMI (Khanna et al., 2024). This is in line with recent reports showing indications that body fat and in particular fat localizations within the body have a different impact on metabolic disorders such as diabetes, hypertension, and dyslipidemia, and on coronary heart disease and systemic inflammation (Khanna et al., 2024; Neeland et al., 2024).

Furthermore, HCC was strongly negatively associated with high-density lipoprotein (HDL), and positively associated with triglycerides, which are different types of lipids in blood. This suggests that higher cortisol concentrations reduce the “good” cholesterol HDL that helps to remove other forms of cholesterol and at the same time elevates the level of triglycerides, which are risk factors for arteriosclerosis and heart diseases (Melamed et al., 2006; Sharrett et al., 2001; Soppert et al., 2020; Toker et al., 2012).

However, the strongest positive correlations were found between HCC and burnout symptoms such as exhaustion, psychosocial stress, and high work demands. Increase of cortisol is not restricted to occupational situations and results in insufficient recovery, as well as reduction of psychological well-being and quality of life. Note, due to an exploratory approach the correlations were not adjusted for multiple testing. After Bonferroni correction, only the correlations HCC with HDL and HCC with OLBI remained significant.

### 4.2 Moderator analyses: primary outcomes

We found that the HCC – burnout association was moderated both by age and the work ability, explaining more than 33% of the variance. In particular, the results showed that the regression was significant for participants with low work ability, mainly in middle-aged and older subgroups, whereas it disappeared in the subgroups defined by good or very good work ability. This is plausible as high work ability indicates a match between an individual and his job. In contrast to this a mismatch reduces work ability (Ilmarinen, 2006). Interestingly, the pattern of results was nearly the same for both the OLBI and MBI-D, suggesting a convergent validity of the burnout measures as the correlation between both was substantial (*r* = .71, *p* < .001). Furthermore, the HCC – burnout association was moderated by chronic stress assessed by SSCS and differed depending on age. The regression model explained a considerable portion (about 44%) of the variance. The HCC – burnout association was strong in all age groups when the participants experienced high levels of chronic stress. Moderate levels of chronic stress affected the association in middle-aged and older adults, whereas younger working adults were not affected. Expectedly, lower levels of chronic stress eliminated the HCC – OLBI association.

Emotional instability (neuroticism) together with age was found to moderate the HCC – burnout association. This personality trait plays a crucial role in development of burnout (Bianchi, 2018), suggesting that individuals with high levels of worry, fear, anxiety, guilt, and depressed mood are likely to respond to stressors with burnout symptoms due to lower stress resilience and this seems to increase with age.

Finally, depression symptoms assessed by the BDI and age explained about 35% of the total variance and moderated the HCC – burnout association. Specifically, BDI scores higher than 7, indicating a mild form of depression, moderated this association. However, the interaction between BDI and age showed only a trend. This suggests that HCC – OLBI association is affected by depressive symptoms regardless of age.

Taken together, the primary self-reported measures that evaluate past and current physical and mental ability to work and matching between individuum and his work, chronic stress level, neuroticism, depression as well as chronological age substantially affected the burnout symptoms. The associations were generally stronger with increasing age. While the relationship between low work ability, stress, neuroticism, and self-reported burnout measures (mainly MBI-GS) has been documented in the literature (Bianchi, 2018; Schouteten, 2016; Sahlin et al., 2014; Wu et al., 2006), the effect of the hair cortisol level as an index of long-term stress and the interaction with age has not been reported so far.

### 4.3 Moderator analyses: biological markers

To ensure health, coordinated physiological systems should be in balance. Long-term stress can imbalance these systems. As a result of a chain reaction, one or more physiological systems can therefore collapse. According to the allostatic load model, imbalance of physiological systems can contribute to burnout (Bärtl et al., 2022; Bellingrath et al., 2009; Juster et al., 2010). In particular, the release of cortisol in response to chronic stress is known to modulate immune cell populations (Aquino-Acevedo et al., 2022; Segerstrom and Miller, 2004). Thus, in the present study we selected some relevant immunological markers to assess whether the HCC – burnout association is moderated by age and these biomarkers. First, T cells as a major component of cellular immunity (Khedri et al., 2020, for review) and changes of T cell concentrations in stress situations have been reported previously (Aquino-Acevedo et al., 2022; Khedri et al., 2020; Kiecolt-Glaser et al., 1986; Maydych et al., 2017). The activation of the HPA axis by stress affects the T cell activity as there is bidirectional communication between the T cells and HPA axis by the release of cortisol (Silverman et al., 2005; McEwen, 1998). Moreover, the percentage and absolute number of the T cells change with age, which leads to a reduced immune response in older individuals and increased inflammation (Li et al., 2019). Indeed, long-term activation of T cells and HPA axis by stress was associated with sustained inflammation. This could be the reason for stronger association between HCC and burnout symptoms induced by inflammatory processes with increasing age. Consistent with this, our results show that the HCC – burnout association was substantially moderated by T cell concentration and age for high concentrations in middle-aged and older working individuals: there was a positive HCC – burnout association for high number of T cells and a negative association for low number of T cells. This pattern was similar for OLBI and MBI-D and all age groups, but it was stronger in middle-aged and older individuals compared to younger ones.

Next, we focused on the CD4/CD8 ratio that defines immune capabilities affecting health status (Garrido-Rodríguez et al., 2021). CD4^+^ T cells modulate the immune response against infections, while CD8^+^ T cells are capable to eliminate cancer cells or cells infected with viruses or bacteria (Sun et al., 2023). Thus, the CD4/CD8 ratio can provide information on how efficient the immune system works. Low or inverted ratios (< 1) indicate a compromised immune system or immunosenescence (Yan et al., 2024)). Low CD4/CD8 ratio was also shown to correlate with severity of major depressive disorder (Zhou et al., 2022) and is part of the immune risk profile (IRP; Wikby et al., 2008). On the other hand, the CD4/CD8 ratio raises during immune aging (Bröde et al., 2023). Our results showed that the HCC – burnout association was substantial for a lower CD4/CD8 ratio in the whole sample. With increasing CD4/CD8 ratios the association was significant in middle-aged and older individuals, whereas high CD4/CD8 ratios moderated the HCC-OLBI associations in the oldest group only, though the high order interaction did not reach significance.

Proinflammatory cytokines IL-6 and TNF-α showed a similar pattern, as both interacted with age and moderated the HCC – burnout association for lower concentrations in older age. Finally, the proinflammatory cytokine IL-18, which promotes the activity of the innate immune system, showed an interaction with age, and moderated the HCC – OLBI association: the negative relationship for young adults and high concentrations reversed and yielded positive relationship for low IL-18 concentrations in older adults. This indicates a concentration- and age-driven dynamic interaction with cytokines in respect to the HCC – burnout relationship. Overall, inflammatory cytokines analyzed in the study were often evaluated in context of psychiatric disorders such as major depression. Meta-analyses indicated an increase of inflammatory biomarkers such as IL-6, IL-18 in depression but regarding TNF-α there were inconsistent reports (Milaneschi et al., 2021; Yuan et al., 2019). At a cellular level, changes with TNF-α induce release of glutamate by activated microglia and leads to excitotoxic damage in the surrounding neurons (Takeuchi et al., 2006). However, in our study the inflammatory cytokines were not correlated with BDI scores, presumably due to low depressive symptoms and a small number of participants with the cytokine measures.

While the results of the primary self-reported outcomes are clear cut for work ability, chronic stress level, neuroticism and depressive symptoms, the findings including the immunological parameters are less intuitive. The strongest positive associations were observed for high concentrations of T cells that increased with age. The ratio CD4/CD8 affected the HCC- OLBI association in the direction that lower CD4/CD8 ratio increased the relationship in all age groups, while increased ratios boosted the HCC-OLBI association in older adults only. This seems plausible as CD4/CD8 ratio is related to cellular immunosenescence and together with persistent inflammation, are known to be involved in the process of compromised aging (Garrido-Rodríguez et al., 2021). Additionally, stress-related cortisol release inhibits telomerase activity both in CD4 and CD8 T cells (Choi et al., 2008). Maydych et al. (2017) reported a reduction of CD4 and CD8 T cell subsets during stressful examination periods in students and a subsequent increase after the stress period. An inverse pattern was observed in the study for proinflammatory cytokines IL-6 and TNF-α, as there was an increase of concentrations during the examination phase and a reduction after the exam period was finished. Our analysis revealed that the HCC – OLBI association was stronger for lower IL-6 and TNF-α concentrations, mainly in older adults. The same was true for IL-18, but the pattern clearly reversed between younger and older adults. No moderation of the HCC – burnout association by age was found for monocytes, granulocytes, B cells, NK cells, and NK-T cells as well as for metabolic parameters used for assessing the allostatic load (such as Hb, CRP, HDL, LDL, or Triglycerides), suggesting that not all biomarkers used for computing the allostatic load have a strong impact on the link between HCC and burnout although HCC may be strongly correlated with some of them (e.g., HDL).

### 4.4 Limitations

There are several limitations of the study that should be acknowledged. First, the group of working participants, who met a minimum hair length of 3 cm and were willing to donate a hair sample, reflected a subsample of 199 working individuals out of 588. In the most cases men had an insufficient hair length to be analyzed regarding cortisol concentration. This led to a high portion of women (84.6%). This precluded the inclusion of sex as factor in the analysis and restricted the data interpretation rather to the female group. Second, only 102 of the 199 eligible participants have been assessed regarding proinflammatory cytokines, due to technical problems when measuring the cytokines. Consequently, while the subgroups with and without proinflammatory cytokines data did not differ in mean age, there was a slightly higher ratio of female participants in the latter group. Third, the participants belonged to a relatively healthy working population and were not selected regarding a particular level of stress or burnout symptoms. This limits the generalizability to normal working populations and excludes clinical groups. Third, we used several questionnaires to evaluate burnout, depression, level of stress, or personality traits. Questionnaires have certain disadvantages such as low power or social desirability that can induce misstatements. Ideally, objective measurements such as biomarkers or computerized tests should be used to quantify these constructs. Nevertheless, there is still no alternative to questionnaires for measuring parameters such as personality, attitudes or lifestyle. The designers of the questionnaires are endeavoring to establish an instrument that uses several positive or negative formulated questions and alternative formulations to capture constructs in a valid way. Furthermore, although the questionnaires are not able to capture the information with 100% reliability, they provide at least a rough quantification of the parameters. Consequently, our study aims to establish biomarkers to better assess the risk of burnout and to help diagnosing burnout in a more valid way. Fourth, we identified 7 persons with moderate depression (3.2%) and 3 persons with severe depression (1.4%) according to the BDI scoring. This may potentially affect the results as the burnout and depressive symptoms are largely overlapping. Nevertheless, after excluding these participants the results remained unchanged and therefore, we have decided not to exclude them from the analysis as higher depression scores reflect a normal distribution in the general population. Finally, we did not use the typical procedure to compute allostatic load index, because some of the physiological parameters included in this index were not measured in our study (e.g., fibrinogen, d-dimer, or body fat). Instead, several alternative biomarkers were evaluated in context of HHC and burnout.

## 5 Conclusions

Taken together, our study showed a stable relationship between HCC and burnout symptoms in working adults aged between 20 and 65 years. The HCC was affected by psychosocial stress at work and problems to recover after work and resulted in a reduced quality of life. The association HCC – burnout was moderated by age, work ability, chronic stress, emotional instability (neuroticism), and depressive symptoms, but also by immunological parameters such as T cells and proinflammatory cytokines. Additionally, the present study provided cutoff scores that indicate at which level of the parameters the risk for association between HCC and burnout is significantly increased and which age group is particularly susceptible. The findings suggest that HCC is a valid marker to objectively measure cumulative effect of work-related stress and emotional exhaustion and depends on a cascade of personality, psychosocial and biological parameters that appears to be enhanced by age. In future studies, longitudinal changes of the HCC – burnout axis could shed more light on its temporal dynamics as a function of these parameters. In sum, extraction of risk factors for the activation of the HCC – burnout bridges an important gap in deciphering the underpinnings of occupational health across the working lifespan. Our results reflect progress in the risk assessment and diagnosis of occupational burnout.

## CRediT authorship contribution statement

**Patrick D. Gajewski:** Conceptualization, Data curation, Formal analysis, Investigation, Methodology, Project administration, Visualization, Writing – original draft, Writing – review & editing. **Peter Bröde:** Data curation, Formal analysis, Investigation, Validation, Writing – review & editing. **Maren Claus:** Data curation, Formal analysis, Investigation, Writing – review & editing. **Klaus Golka:** Investigation, Methodology, Writing – review & editing. **Jan G. Hengstler:** Funding acquisition, Investigation, Methodology, Resources, Writing – review & editing. **Carsten Watzl:** Data curation, Investigation, Methodology, Resources, Writing – review & editing. **Edmund Wascher:** Data curation, Funding acquisition, Methodology, Project administration, Resources, Supervision, Writing – review & editing. **Stephan Getzmann:** Conceptualization, Data curation, Methodology, Project administration, Supervision, Validation, Writing – review & editing.

## Institutional review Board Statement

The study was conducted in accordance with the Declaration of Helsinki and was approved by the Institutional Ethics Committee of IfADo (approval number: A93-1). Informed consent was obtained from all subjects involved in the study as detailed in the published study protocol (Gajewski et al., 2022).

## Funding

The *Dortmund Vital Study* is funded by the institute's budget. Thus, the study design, collection, management, analysis, interpretation of data, writing of the report, and the decision to submit the report for publication is not influenced or biased by any sponsor.

## Declaration of competing interest

The authors declare that they have no interests to declare.

## Data Availability

The data presented in this study are available on request from the corresponding author.
